# Development and Validation of a Nomogram for Predicting Long-Term Net Adverse Clinical Events in High Bleeding Risk Patients Undergoing Percutaneous Coronary Intervention

**DOI:** 10.31083/RCM25352

**Published:** 2025-01-17

**Authors:** Junyan Zhang, Zhongxiu Chen, Ran Liu, Yuxiao Li, Hongsen Zhao, Yanning Li, Minggang Zhou, Hua Wang, Chen Li, Li Rao, Yong He

**Affiliations:** ^1^Department of Cardiology, West China Hospital of Sichuan University, 610041 Chengdu, Sichuan, China; ^2^Information Center of West China Hospital, Sichuan University, 610041 Chengdu, Sichuan, China; ^3^Department of Nursing, West China Hospital of Sichuan University, 610041 Chengdu, Sichuan, China

**Keywords:** percutaneous coronary intervention, high bleeding risk, nomogram, prognosis, prediction model, internal validation

## Abstract

**Background:**

Patients with a high risk of bleeding undergoing percutaneous coronary intervention (PCI-HBR) were provided consensus-based criteria by the Academic Research Consortium for High Bleeding Risk (ARC-HBR). However, the prognostic predictors in this group of patients have yet to be fully explored. Thus, an effective prognostic prediction model for PCI-HBR patients is required.

**Methods:**

We prospectively enrolled PCI-HBR patients from May 2022 to April 2024 at West China Hospital of Sichuan University. The cohort was randomly divided into training and internal validation sets in a ratio of 7:3. The least absolute shrinkage and selection operator (LASSO) regression algorithm was employed to select variables in the training set. Subsequently, a prediction model for 1-year net adverse clinical events (NACEs)-free survival was developed using a multivariable Cox regression model, and a nomogram was constructed. The outcome of the NACEs is defined as a composite endpoint that includes death, myocardial infarction, ischemic stroke, and Bleeding Academic Research Consortium (BARC) grade 3–5 major bleeding. Validation was conducted exclusively using the internal validation cohort, assessing the discrimination, calibration, and clinical utility of the nomogram.

**Results:**

This study included 1512 patients with PCI-HBR, including 1058 in the derivation cohort and 454 in the validation cohort. We revealed five risk factors after LASSO regression, Cox regression, and clinical significance screening. These were then utilized to construct a prognostic prediction nomogram, including chronic kidney disease, left main stem lesion, multivessel disease, triglycerides (TG), and creatine kinase-myocardial band (CK-MB). The nomogram exhibited strong predictive ability (the area under the curve (AUC) to predict 1-year NACE-free survival was 0.728), displaying favorable levels of accuracy, discrimination, and clinical usefulness in the internal validation cohort.

**Conclusions:**

This study presents a nomogram to predict 1-year NACE outcomes in PCI-HBR patients. Internal validation showed strong predictive capability and clinical utility. Future research should validate the nomogram in diverse populations and explore new predictors for improved accuracy.

**Clinical Trial Registration:**

The data for this study were obtained from the PPP-PCI registry, NCT05369442 (https://clinicaltrials.gov/study/NCT05369442).

## 1. Introduction

According to current guidelines, percutaneous coronary intervention (PCI) is a crucial therapeutic method for patients who have experienced an acute myocardial infarction and can dramatically enhance outcomes and lower in-hospital mortality in people with coronary artery disease [1]. However, bleeding represents a frequent side effect of antiplatelet therapy following PCI and can raise the rate of severe cardiovascular events as well as the cost of treatment [2]. According to recent clinical studies, PCI patients with a high bleeding risk (HBR) often have a reported incidence among PCI patients in clinical practice of around 40% [3, 4].

Although there are currently several prognostic models and scoring systems for patients undergoing PCI, such as the CRUSADE [5] and thrombolysis in myocardial infarction (TIMI) [6] scores, these systems do not specifically target the HBR population. Existing prediction models for patients with a HBR following PCI (PCI-HBR) primarily focus on bleeding events as the primary outcome [7, 8]. Therefore, there remains a lack of dedicated prognostic models specifically tailored to this patient population, which could provide better guidance for clinical decision-making.

Thus, we prospectively derived and validated a prediction model for long-term net adverse clinical events (NACEs) in PCI-HBR patients and constructed a nomogram. This model aims to assist cardiologists in identifying high-risk patients early and creating individualized treatment plans.

## 2. Methods

We followed the TRIPOD [9] (Transparent Reporting of a Multivariable Prediction Model for Individual Prognosis or Diagnosis) statement for reporting the development and validation of a multivariable prediction model. This study was approved by the Biomedical Research Ethics Committee of West China Hospital (No.2022-269). All patients provided informed consent for the procedure and the subsequent data collection and analysis for research.

### 2.1 Study Population

The study population consisted of all patients with PCI-HBR at West China Hospital, Sichuan University. The derivation cohort and internal validation cohort of this study consisted of data collected from PCI-HBR patients from May 2022 to April 2024. The inclusion criteria were based on the Academic Research Consortium for high bleeding risk (ARC-HBR) criteria (**Supplementary Table 1**) [10]. Patients are considered at HBR if at least one major or two minor criteria are met. Patients who refused follow-up were excluded from the study.

### 2.2 Data Collection

Demographic and clinical data were systematically collected, encompassing patient characteristics, vital signs, and medical history. Detailed procedural information and echocardiographic parameters were also documented. Laboratory assessments included hematological parameters, biochemical markers, and cardiac markers, alongside evaluations of liver function tests, lipid profiles, renal function, and coagulation parameters. Furthermore, inflammatory markers and glycemic parameters were measured to provide a comprehensive overview of the patients’ health status.

### 2.3 Study Outcome

The primary endpoint of this study was a NACE, defined as a composite endpoint comprising all-cause mortality, recurrent myocardial infarction, ischemic stroke, and BARC 3–5 major bleeding. Each specific endpoint adheres to the definitions established by the ARC [11].

NACEs were selected as the primary endpoint because they encompass both ischemic and bleeding events, providing a more comprehensive assessment of the prognosis for these patients. This decision is based on the understanding that individuals in this cohort have a higher risk of both ischemic and bleeding complications compared to non-HBR patients.

### 2.4 Model Derivation

Various parameters, including patients’ baseline demographic information, comorbidities, laboratory test data, and echocardiographic results, were considered candidate variables for constructing the prediction model. The least absolute shrinkage and selection operator (LASSO) algorithm was utilized to identify the most significant predictive features from the variables within the derivation cohort [12, 13]. For multivariable analysis, we utilized L1-penalized least absolute shrinkage and selection regression, including 10-fold cross-validation for internal validation. This LASSO–Cox regression model penalizes the absolute size of a regression model’s coefficients according to the value of λ. Only the strongest predictors remain in the model due to greater penalties, which causes the estimates of weaker components to shrink toward zero. The minimum (λ min) was used to choose the most predictive factors. Next, we utilized the multivariate Cox regression method to construct a prognostic model incorporating these indicators. All analyses were performed using R software (version 4.1.2, R Foundation for Statistical Computing, Beijing, China). A *p*-value less than 0.05 was deemed statistically significant for each statistical analysis.

### 2.5 Internal Validation

The predictive performance was quantified from the three perspectives of discrimination, calibration, and clinical utility. Time-dependent receiver operating characteristic curve (ROC) analysis was applied to assess the discrimination of the model, and the corresponding C-index was calculated. Accuracy was evaluated by plotting the calibration curve of 1000 bootstrap samples. We plotted the calibration curves for 1-year NACE-free survival to compare the concordance between the predicted survival probability calculated by the nomogram and the actual survival probability of the patients. Finally, the decision curve analysis (DCA) method was used to estimate the potential clinical value of the nomogram with quantitative analysis of the net benefits being applied at different threshold probabilities. This method is used to evaluate the benefits of a model and incorporate the series of patients’ preferences for the risks of undertreatment and overtreatment to promote more appropriate model selection and inform decision usage [14, 15].

### 2.6 Statistical Analysis

We employed the K-nearest neighbors (KNN) method to impute missing binary and continuous variables. Descriptive statistics were utilized to provide an overview of the basic characteristics and comorbidities of the study population. Continuous variables are described as mean ± standard deviation (SD) if they follow a normal distribution; otherwise, they are presented as median and interquartile range (IQR). Categorical variables are reported as counts and percentages. When transforming a continuous variable into a binary variable, the threshold selection is based on guidelines, consensus, and clinical experience. Statistical significance is defined as a two-sided *p*-value < 0.05.

Statistical analyses were performed using R 4.1.2. The LASSO regression was carried out using the R package “glmnet” statistical program (R Foundation).

## 3. Results

### 3.1 Baseline Characteristics

**Fig. 1.**
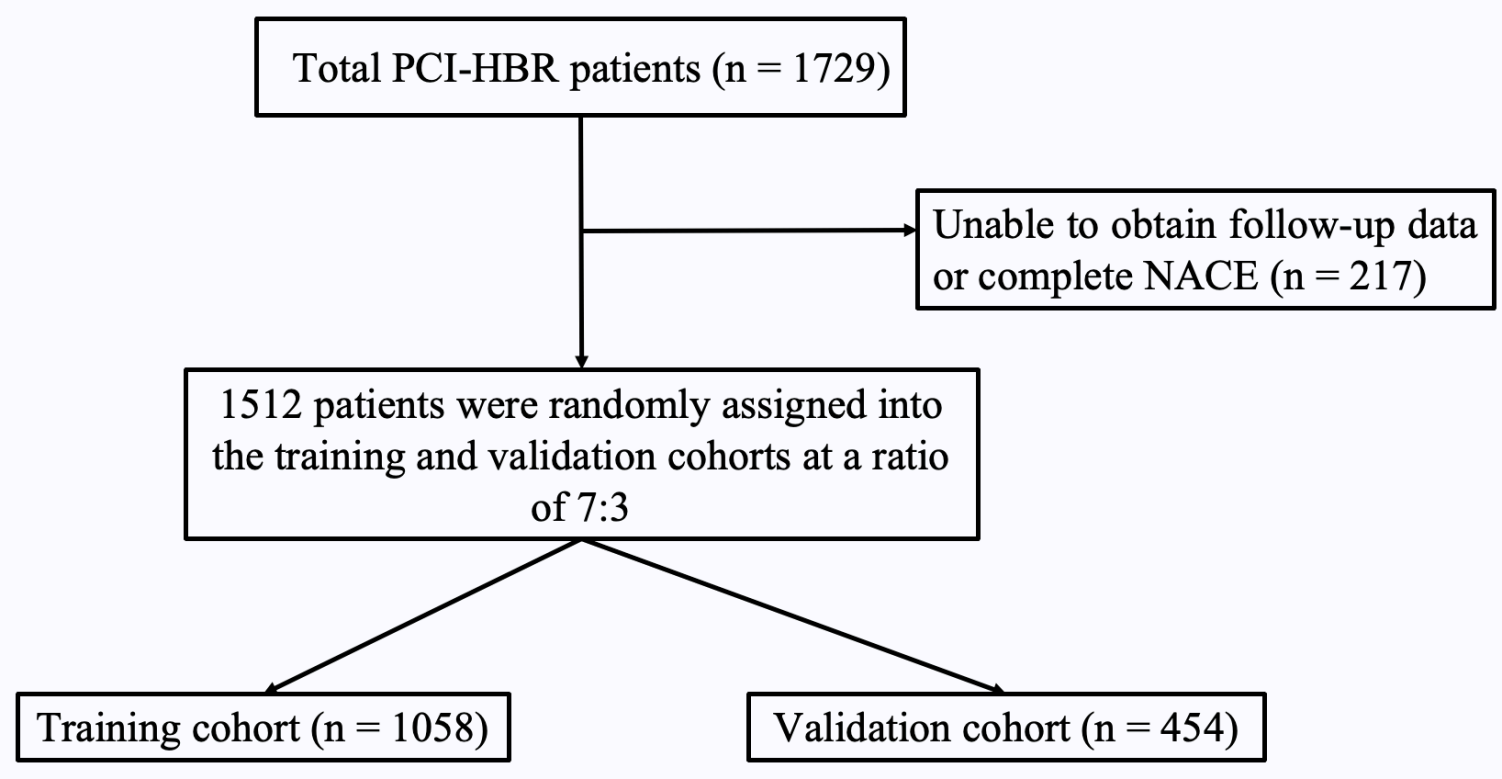
**Study flowchart**. PCI-HBR, percutaneous coronary intervention–high bleeding risk; NACE, net adverse clinical event.

According to the inclusion and exclusion criteria, 1512 patients with PCI-HBR from May 2022 to April 2024 from whom follow-up data could be obtained were eligible for this study, including 1058 in the training cohort and 454 in the validation cohort [16]. Fig. 1 presents a comprehensive flowchart outlining the detailed research process.

The study encompassed 1512 participants with a median age of 70 years (interquartile range (IQR): 60–77), predominantly male (71.6%). The median weight was 63 kg (IQR: 55–70), and the median height was 163 cm (IQR: 156–169). Cardiac function parameters included a median left ventricular ejection fraction (LVEF) of 48.9% (IQR: 46–54) and a median ejection fraction (EF) of 61% (IQR: 50–68). Hematological measures showed a median hemoglobin level (Hb) of 124 g/dL (IQR: 109–139) and a median platelet (PLT) count of 180 × 10^9^/L (IQR: 137–224). The median B-type natriuretic peptide (BNP) level was 611 pg/mL (IQR: 151–2305).

Comorbid conditions were prevalent, with 60.1% of participants having hypertension, 41.0% having diabetes mellitus, and 29.6% hyperlipidemia. Acute coronary syndrome was present in 51.1% of the cohort, while atrial fibrillation and chronic kidney disease were observed in 8.2% and 22.6% of participants, respectively. Coronary artery disease was notably prevalent, with 43.8% exhibiting multivessel disease and 23.1% having chronic total occlusion. Interventional procedures such as femoral access (1.2%) and intra-aortic balloon pump use (3.4%) were relatively rare in this population. The training and internal validation cohorts were randomly split at a 7:3 ratio, and the two groups had no statistical differences in almost all of the baseline data (Table 1, **Supplementary Table 2**).

**Table 1.** **Patient demographics and baseline characteristics**.

Characteristic	Cohort			*p*-value
	Overall (n = 1512)	Training cohort (n = 1058)	Internal validation cohort (n = 454)	
Age	70 (60, 77)	70 (60, 77)	70 (60, 77)	0.884
Male	1082 (71.6%)	759 (71.7%)	323 (71.1%)	0.815
Heart rate	77 (69, 87)	78 (69, 87)	76 (68, 87)	0.355
Systolic blood pressure	131 (118, 146)	131 (118, 146)	132 (117, 146)	0.972
Diastolic blood pressure	77 (70, 85)	78 (70, 86)	77 (70, 85)	0.792
Acute coronary syndrome	772 (51.1%)	534 (50.5%)	238 (52.4%)	0.487
Hypertension	908 (60.1%)	648 (61.2%)	260 (57.3%)	0.148
Diabetes mellitus	620 (41.0%)	438 (41.4%)	182 (40.1%)	0.635
Hyperlipidemia	447 (29.6%)	315 (29.8%)	132 (29.1%)	0.785
Atrial fibrillation	124 (8.2%)	94 (8.9%)	30 (6.6%)	0.139
Ischemic stroke	97 (6.4%)	67 (6.3%)	30 (6.6%)	0.841
Hemorrhagic stroke	8 (0.5%)	5 (0.5%)	3 (0.7%)	0.703
Chronic kidney disease	342 (22.6%)	235 (22.2%)	107 (23.6%)	0.563
FFR-guided PCI	67 (4.4%)	47 (4.4%)	20 (4.4%)	0.974
Transfer to CCU	200 (13.2%)	149 (14.1%)	51 (11.2%)	0.134
Left main stem lesion	309 (20.4%)	230 (21.7%)	79 (17.4%)	0.055
Multivessel disease	662 (43.8%)	468 (44.2%)	194 (42.7%)	0.589
Intra-aortic balloon pump	51 (3.4%)	40 (3.8%)	11 (2.4%)	0.180
Chronic total occlusion	350 (23.1%)	251 (23.7%)	99 (21.8%)	0.418
Left ventricle (mm) *	48.9 (46.0, 54.0)	49.0 (46.0, 54.0)	48.0 (46.0, 54.0)	0.102
Ejection fraction (%) *	61 (50, 68)	61 (50, 68)	62 (51, 69)	0.243
Hemoglobin (g/L)	124 (109, 139)	123 (109, 139)	125 (110, 138)	0.729
Platelet account (×10^9^/L)	180 (137, 224)	180 (137, 223)	178 (136, 227)	0.848
Cardiac troponin T (µg/L)	24 (12, 305)	24 (11, 360)	27 (13, 219)	0.775
Myoglobin (ng/mL)	41 (28, 86)	42 (28, 88)	40 (27, 74)	0.086
CK-MB (U/L)	2 (1, 4)	2 (1, 4)	2 (1, 4)	0.385
NT-pro-BNP (ng/L)	611 (151, 2305)	643 (153, 2360)	546 (150, 2139)	0.455
eGFR (mL/min × 1.73 m^2^)	65 (42, 85)	65 (41, 85)	65 (44, 86)	0.292
HbA1c (%) *	6.60 (5.90, 8.40)	6.60 (5.90, 8.40)	6.60 (5.90, 8.30)	0.976

Median (IQR); n (%). FFR-guided PCI, fractional flow reserve-guided percutaneous coronary intervention; CCU, coronary care unit; CK-MB, creatine kinase-myocardial band; NT-pro-BNP, N-terminal pro-b-type natriuretic peptide; eGFR, estimated glomerular filtration rate; HbA1c, hemoglobin A1c. *The marked variables indicate those imputed, with 100 imputations for left ventricle and ejection fraction and 198 imputations for HbA1c.

### 3.2 Model Derivation

**Fig. 2.**
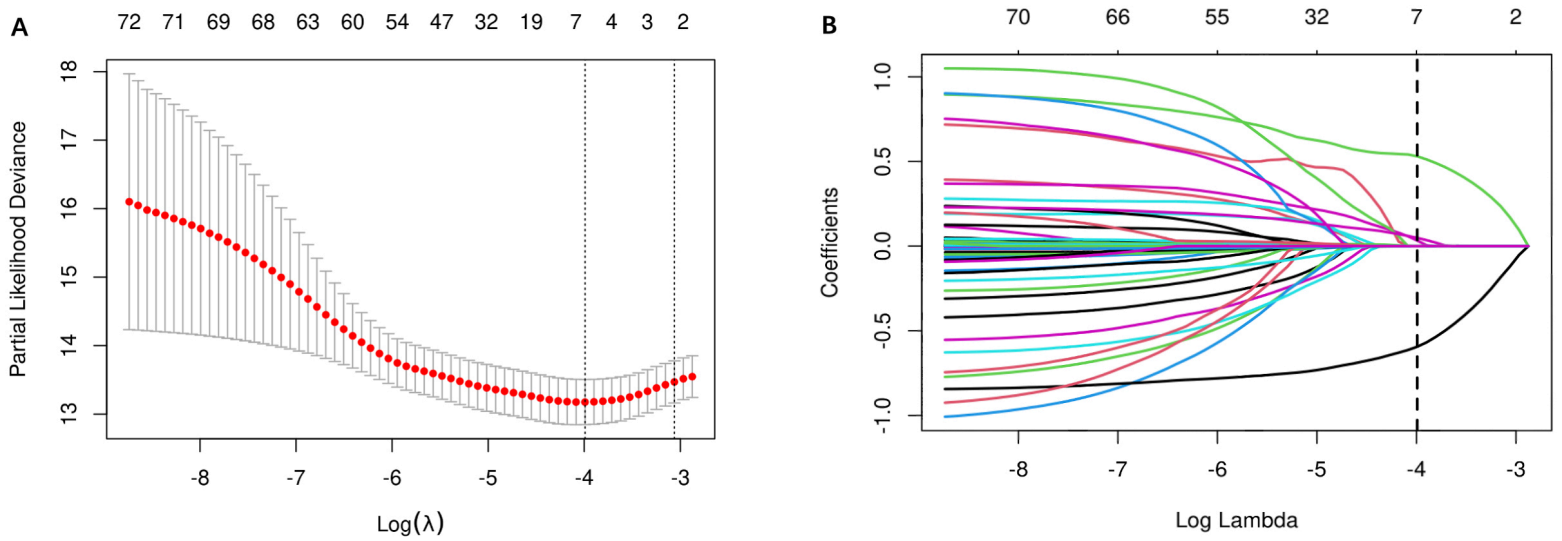
**Lasso regression analysis**. (A) Lasso regression cross-validation plot. (B) Lasso regression coefficient path plot.

The initial model included a range of candidate predictors, such as sex, age, heart rate, blood pressure, weight, height, acute coronary syndrome, hypertension, diabetes mellitus, and hyperlipidemia, among others, encompassing patient demographics, baseline comorbidities, laboratory tests, echocardiographic parameters, and interventional procedure-related metrics, totaling 65 variables (**Supplementary Table 2**). LASSO regression analysis on the training cohort reduced these variables to five potential predictors. The cross-validated error plot and regression coefficient path plot for the LASSO regression model are presented in Fig. 2. The resulting model, which is highly regularized and parsimonious, includes only five variables and achieves a cross-validated error within one standard error of the minimum.

**Fig. 3.**
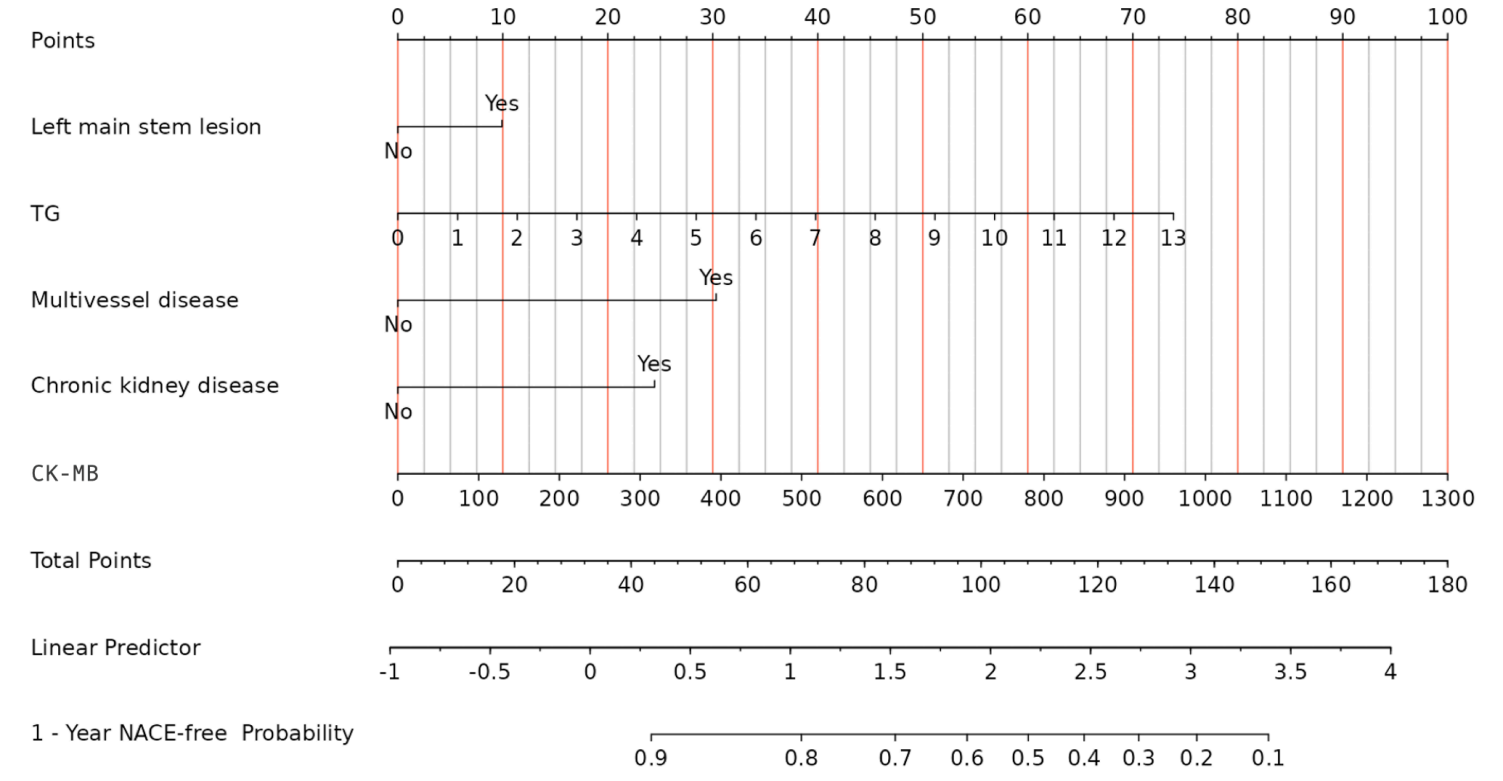
**Nomogram prediction model**. TG, triglycerides; CK-MB, creatine kinase-myocardial band; NACE, net adverse clinical event.

**Fig. 4.**
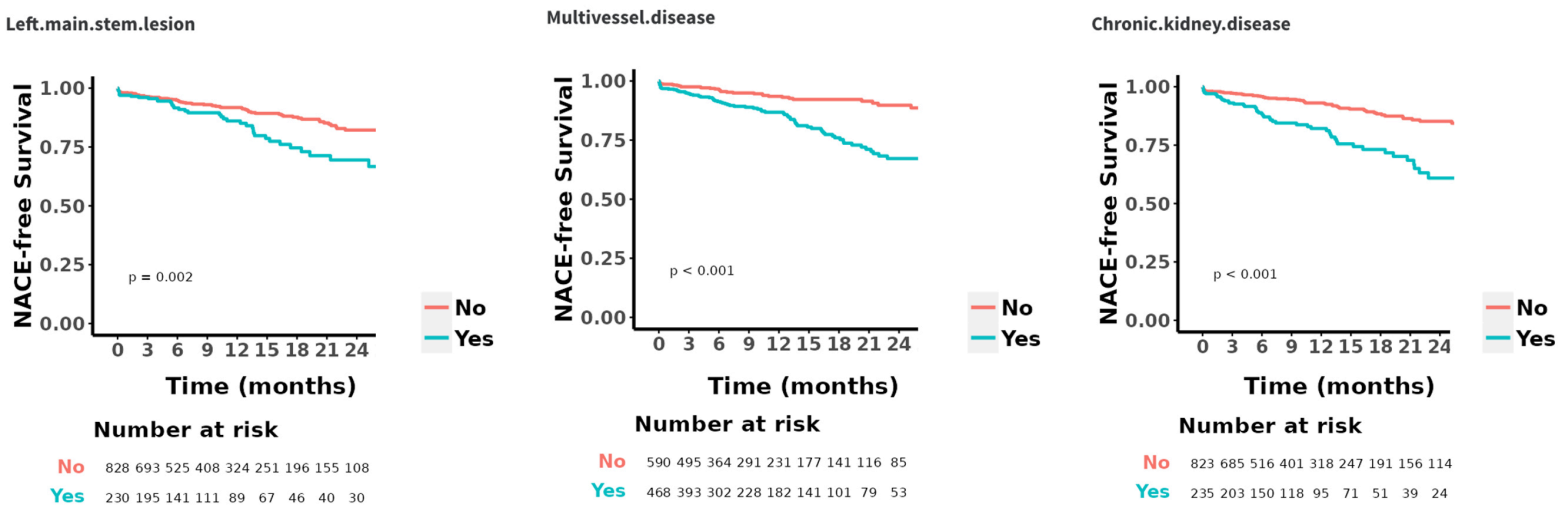
**Kaplan–Meier curves for categorical variables in the final prediction model**. NACE, net adverse clinical event.

Additional multivariate Cox analyses were performed on the training cohorts, with the results in Table 2. The Cox model incorporated five independent predictors (chronic kidney disease, left main stem lesion, multivessel disease, triglycerides (TGs), and creatine kinase-myocardial band (CK-MB)) and was developed into a user-friendly nomogram as illustrated in the following figure (Fig. 3). The Kaplan–Meier curves for the three categorical variables included in the final model are presented in Fig. 4.

**Table 2.** **Results of multivariate Cox regression for the training cohort**.

Characteristic	HR	95% CI	*p*-value
Multivessel disease	2.44	1.59, 3.70	<0.001
Chronic kidney disease	2.04	1.39, 2.99	<0.001
Left main stem lesion	1.34	0.89, 2.00	0.158
TG	1.18	1.04, 1.34	0.013
CK-MB	1.00	1.00, 1.00	<0.001

HR, hazard ratio; CI, confidence interval; TG, triglycerides; CK-MB, creatine kinase-myocardial band.

### 3.3 Model Validation

**Fig. 5.**
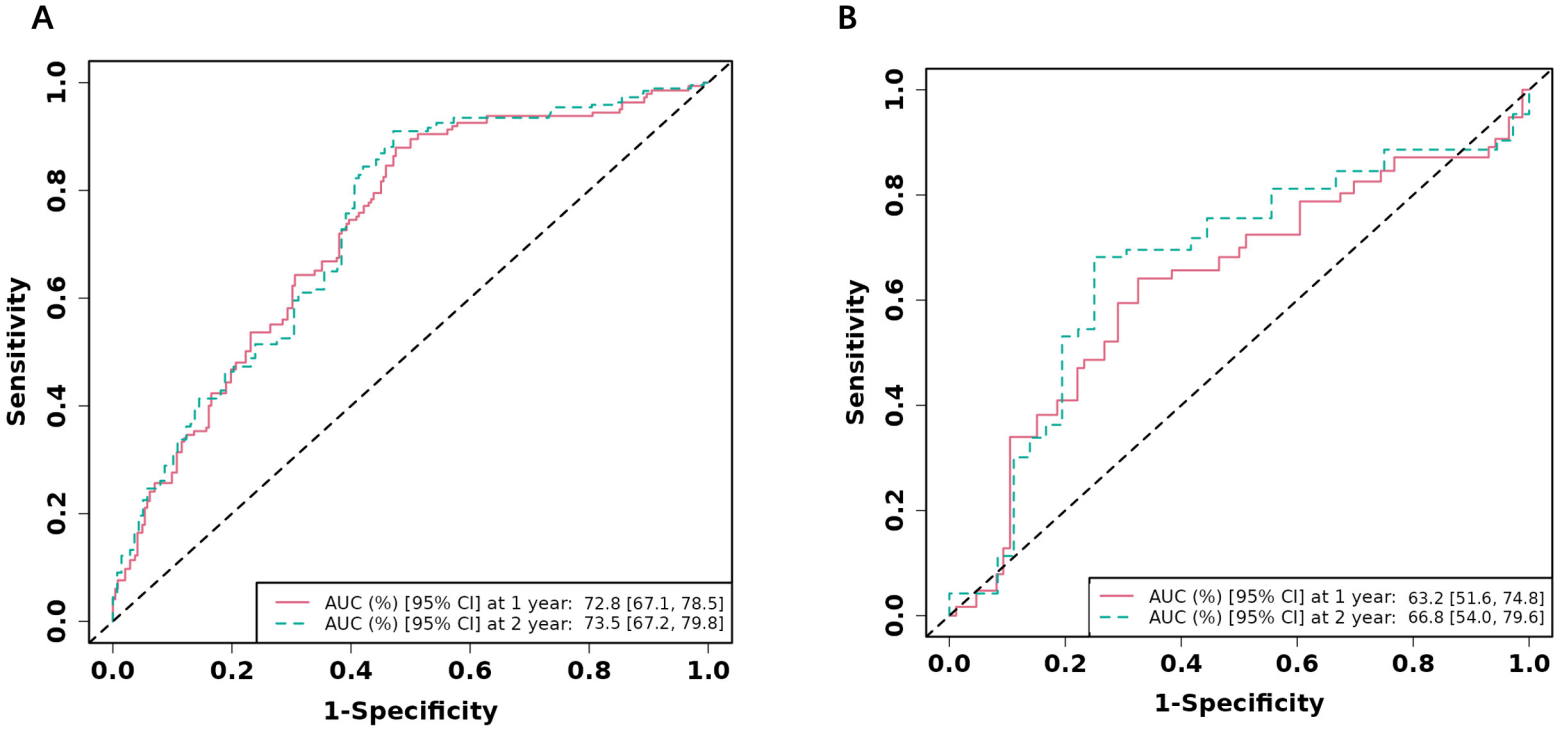
**Receiver operating characteristic (ROC) curves**. ROC curves of the nomogram prediction model in training cohort (A) and internal validation cohort (B). AUC, area under the curve.

The time-dependent receiver operating characteristic (ROC) curves for predicting 1-year NACE-free survival demonstrated favorable discrimination in both the derivation and internal validation cohorts (Fig. 5).

**Fig. 6.**
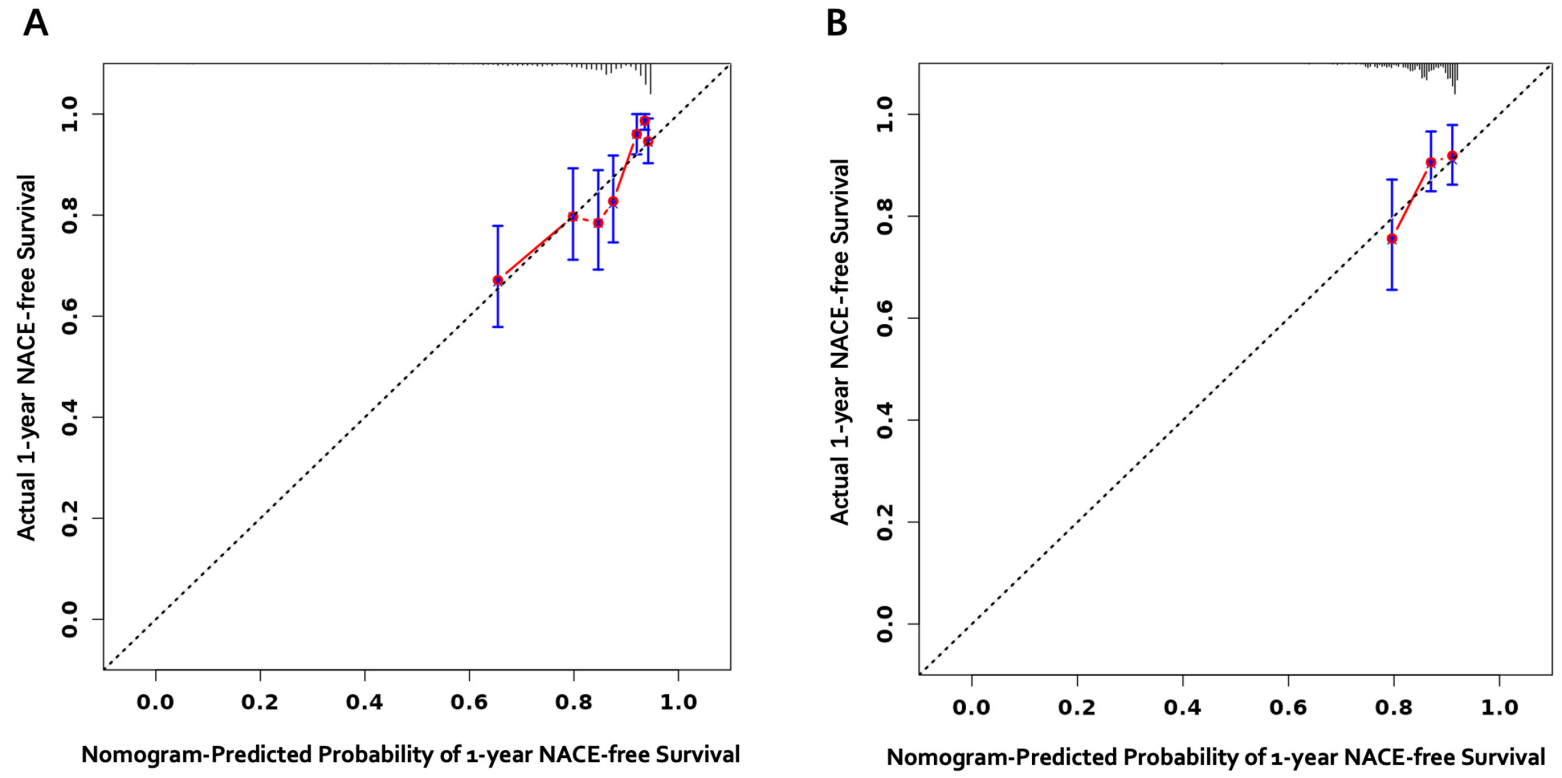
**Calibration curves**. Calibration curves of the nomogram prediction model in 1-year net adverse clinical event (NACE)-free survival in training cohort (A) and internal validation cohort (B).

The calibration plots of the nomogram in derivation and internal validation cohorts are shown in the following figures (Fig. 6), demonstrating a strong correlation between observed and predicted risk. The results indicated that the original nomogram remained appropriate for use in the validation sets, with the calibration curve of this model being relatively close to the ideal curve, suggesting that the predicted outcomes were consistent with the actual findings.

**Fig. 7.**
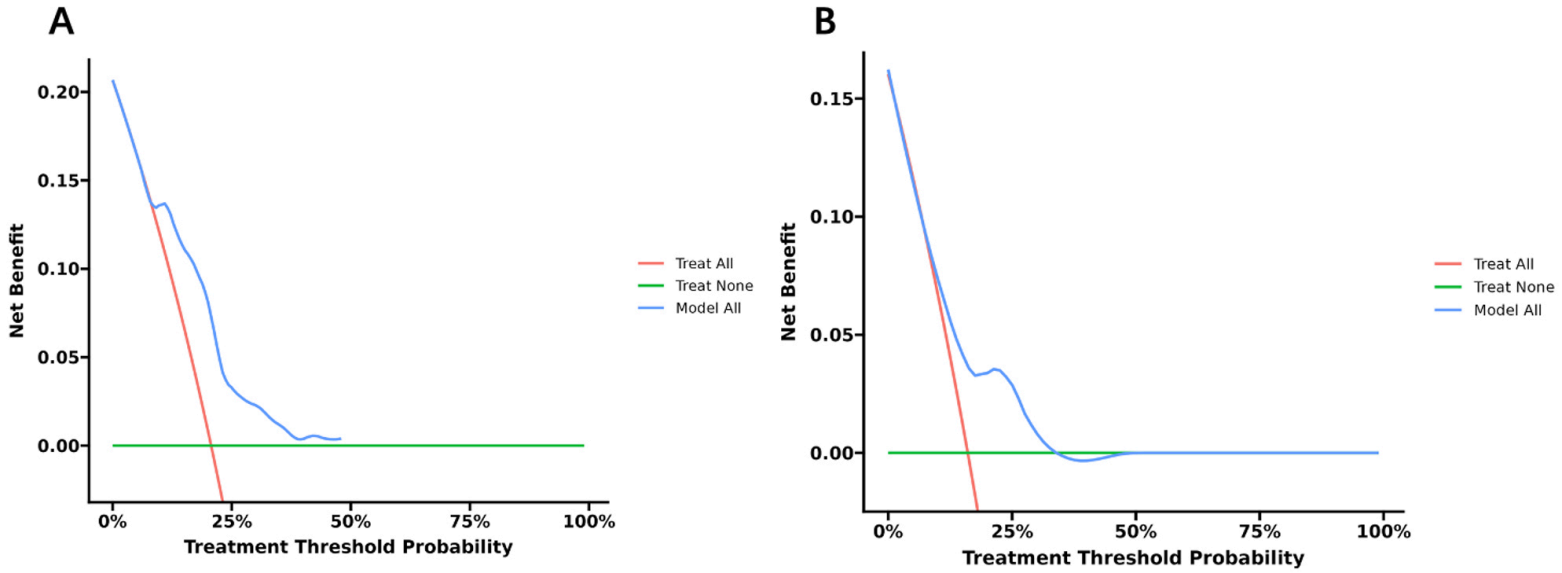
**Decision curve analysis (DCA) curves**. DCA curves of the nomogram prediction model in 1-year NACE-free survival in the training cohort (A) and internal validation cohort (B). NACE, net adverse clinical event.

Fig. 7 presents the DCA curves associated with the nomogram. This study demonstrates that the nomogram provides considerable net benefits for clinical use, as evidenced by the DCA curve. In the DCA curve, the horizontal and vertical axes represent the threshold probability and net benefit, respectively, with the lines between the axes displaying the benefit of different predictive variables. The DCA curves indicate that if the threshold probability is between 10% and 50%, using this nomogram in the current study to predict 1-year NACEs could offer additional benefits.

## 4. Discussion

With advancements in coronary artery intervention techniques, developments in stent technology, changes in access sites, and gradual control of high ischemic factors such as diabetes and hypertension, there has been a significant decrease in the occurrence of post-PCI ischemic events, such as in-stent thrombosis and restenosis. Conversely, a growing focus has shifted to bleeding events, which have emerged as common complications following PCI. Major bleeding significantly elevates the risks of adverse cardiovascular events, including death, as well as other perioperative complications, thereby escalating treatment costs [2]. Consequently, the early identification of high-risk PCI-HBR patients is paramount. However, there is currently a lack of a universally recognized, concise, convenient, and reliable prognostic model specifically tailored to predict adverse outcomes in PCI-HBR patients.

This study presents, for the first time, a prognostic nomogram for predicting long-term outcomes in PCI-HBR patients based on a cohort of 1512 patients. We included various available clinical indicators, including patient demographics, baseline comorbidities, laboratory tests, echocardiographic parameters, and interventional procedure-related metrics, and utilized LASSO regression for variable selection. Subsequently, a multivariable Cox regression model was employed for further selection, resulting in the construction of the nomogram. Five variables were included in the model: chronic kidney disease, left main stem lesion, multivessel disease, TG, and CK-MB. Through internal validation, this model demonstrated strong predictive capability, satisfactory calibration, discrimination, and clinical utility. We hope this validated nomogram can effectively guide clinicians, enabling early identification of high-risk PCI-HBR patients, facilitating timely interventions, and improving communication with patients and their families regarding their condition.

In fact, some prediction models have been established specifically for PCI-HBR patients [17, 18]. However, their primary objective is to predict major bleeding risk rather than survival outcomes. Additionally, scoring systems such as CRUSADE [5] and TIMI [6] can also be used to indicate the prognosis of PCI patients. However, these models were not specifically developed based on the HBR population, meaning they cannot accurately predict the overall prognosis of PCI-HBR patients. Therefore, we constructed and validated a prognostic prediction model with all-cause mortality as an endpoint specifically for PCI-HBR patients to improve clinical practice treatments.

This study screened and included five variables in the final model: chronic kidney disease, left main stem lesion, multivessel disease, TG, and CK-MB, all of which have good interpretability and prognostic predictive value. Numerous studies have shown that chronic kidney disease (CKD) strongly predicts poor prognosis in PCI patients [19, 20]. In the assessment of bleeding and ischemia, CKD is a unique factor, as it is both a high bleeding risk factor [10] and a high ischemia risk factor [21], playing an extremely important role in the management of PCI patients [22]. As risk factors in the SYNTAX scoring system, left main stem lesion and multi-vessel disease represent the complexity of coronary artery disease and are important prognostic predictors for PCI patients [23, 24]. Similarly, multiple studies have demonstrated elevated CK-MB levels as an independent risk factor for PCI patients [25, 26]. Ioannidis *et al*. [27], through a meta-analysis of seven studies, found a stepwise increase in the risk of death with rising CK-MB levels. Stone *et al*. [28] also found that only CK-MB elevations were independently associated with increased 2-year mortality. The findings of this study align with these previous results. Interestingly, our study used LASSO regression to screen for myocardial injury markers and identified CK-MB rather than cardiac troponin, a more accurate biomarker for myocardial injury. This finding is consistent with the study by Garcia-Garcia *et al*. [29], who pooled data from five contemporary coronary stent trials and one large registry, totaling 13,452 PCI patients, and found that elevated CK-MB levels after PCI were associated with increased mortality. In contrast, cardiac troponin elevation was not independently associated with 1-year mortality. Our conclusion suggests that CK-MB might better predict long-term adverse events in PCI-HBR patients than cardiac troponin. This hypothesis warrants further investigation using larger sample sizes. Additionally, multiple studies have demonstrated a significant association between TG levels and the risk of coronary artery disease [30, 31, 32]. Furthermore, elevated TG levels are significantly correlated with all-cause mortality in patients with coronary artery disease, and this association persisted over the long-term follow-up [33]. Our study also found that high TG levels are an independent risk factor for adverse long-term outcomes in patients with PCI-HBR.

Our study has several limitations that warrant acknowledgment. Firstly, the cohort comprised West China Hospital of Sichuan University patients, which may not reflect the broader population. Additionally, unmeasured confounders could not be accounted for in our model. Therefore, external validation in diverse populations is crucial to confirm the generalizability of our results.

## 5. Conclusions

This study is the first to introduce a novel prognostic nomogram designed to predict 1-year NACE outcomes in PCI-HBR patients, based on a cohort of 1512 individuals. We identified five key variables: chronic kidney disease, left main stem lesion, multivessel disease, TG, and CK-MB. The internal validation of the model demonstrated strong predictive capability, satisfactory calibration, discrimination, and clinical utility. Future studies should focus on externally validating our nomogram across various populations and contexts. Moreover, incorporating new predictors or biomarkers may improve the predictive accuracy of the nomogram, which merits additional exploration.

## Data Availability

The datasets used and analyzed during the current study are available from the corresponding author on reasonable request.

## Abbreviations

PCI, percutaneous coronary intervention; HBR, high bleeding risk; ARC-HBR, the Academic Research Consortium for High Bleeding Risk; AF, atrial fibrillation; CKD, chronic kidney disease; CK-MB, creatine kinase-MB; NT-pro-BNP, N-terminal pro-b-type natriuretic peptide; LASSO, least absolute shrinkage and selection operator.

## Supplementary Material

Supplementary material associated with this article can be found, in the online version, at https://doi.org/10.31083/RCM25352.
