# Supplementary material for: Development and Validation of a Nomogram for Predicting Long-Term Net Adverse Clinical Events in High Bleeding Risk Patients Undergoing Percutaneous Coronary Intervention
Source: Rev Cardiovasc Med. 2025 Jan 17;26(1):25352. doi: 10.31083/RCM25352 (PMC11760544; doi:10.31083/RCM25352)
Supplement: Supplementary file 1 [file 2153-8174-26-1-25352-s1.docx]

**Supplementary Table 1 Inclusion criteria**

| Major | Minor |
| --- | --- |
|  | Age more than 75y |
| Anticipated use of long-term oral anticoagulation |  |
| Severe or end-stage CKD (eGFR <30 mU/min) | Moderate CKD (eGFR 30-59 mU min) |
| Hemoglobin <11 g/dL | Hemoglobin 11-12.9 g/dL for men and 11-11.9 g/dL for women |
| Spontaneous bleeding requiring hospitalization or transfusion in the past 6 mo or at any time, if recurrent | Spontaneous bleeding requiring hospitalization or transfusion within the past 12 mo not meeting the major criterion |
| Moderate or severe baseline thrombocytopenia (platelet count <100x109/L) |  |
| Chronic bleeding diathesis |  |
| Liver cirrhosis with portal hypertension |  |
|  | Long-term use of oral NSAIDs or steroids |
| Active malignancy (excluding nonmelanoma skin cancer) within the past 12 mo |  |
| Previous spontaneous ICH (at any time) Previous traumatic ICH within the past 12 mo Presence of a bAVM Moderate or severe ischemic strokes§ within the past 6 mo | Any ischemic stroke at any time not meeting the major criterion |
| Nondeferrable major surgery on DAPT |  |
| Recent major surgery or major trauma within 30 d before PCI |  |

Abbreviations: bAVM indicates brain arteriovenous malformation; CKD, chronic kidney disease; DAPT, dual antiplatelet therapy; eGFR, estimated glomerular filtration rate; HBR, high bleeding risk; ICH, intracranial hemorrhage; NSAID, nonsteroidal anti-inflammatory drug; and PCI, percutaneous coronary intervention.

**Supplementary Table 2:Patient demographics and baseline characteristics**

| Characteristic | Cohort | | | p-value |
| --- | --- | --- | --- | --- |
|  | Overall, N = 1,512 | Training Cohort, N = 1,058 | Internal Test Cohort, N = 454 |  |
| Male | 1,082 (71.6%) | 759 (71.7%) | 323 (71.1%) | 0.815 |
| Age | 70 (60, 77) | 70 (60, 77) | 70 (60, 77) | 0.884 |
| Current smoking | 260 (17.2%) | 163 (15.4%) | 97 (21.4%) | 0.005 |
| Drinking | 122 (8.1%) | 80 (7.6%) | 42 (9.3%) | 0.269 |
| Hear rate | 77 (69, 87) | 78 (69, 87) | 76 (68, 87) | 0.355 |
| Systolic blood pressure | 131 (118, 146) | 131 (118, 146) | 132 (117, 146) | 0.972 |
| Diastolic blood pressure | 77 (70, 85) | 78 (70, 86) | 77 (70, 85) | 0.792 |
| Weight | 63 (55, 70) | 63 (55, 70) | 63 (56, 71) | 0.334 |
| Hight | 163 (156, 169) | 163 (156, 169) | 164 (158, 168) | 0.323 |
| Acute coronary syndrome | 772 (51.1%) | 534 (50.5%) | 238 (52.4%) | 0.487 |
| Hypertension | 908 (60.1%) | 648 (61.2%) | 260 (57.3%) | 0.148 |
| Diabetes mellitus | 620 (41.0%) | 438 (41.4%) | 182 (40.1%) | 0.635 |
| Hyperlipidemia | 447 (29.6%) | 315 (29.8%) | 132 (29.1%) | 0.785 |
| Atrial fibrillation | 124 (8.2%) | 94 (8.9%) | 30 (6.6%) | 0.139 |
| Ischemic stroke | 97 (6.4%) | 67 (6.3%) | 30 (6.6%) | 0.841 |
| Hemorrhagic stroke | 8 (0.5%) | 5 (0.5%) | 3 (0.7%) | 0.703 |
| Chronic kidney disease | 342 (22.6%) | 235 (22.2%) | 107 (23.6%) | 0.563 |
| Gout | 129 (8.5%) | 94 (8.9%) | 35 (7.7%) | 0.453 |
| Chronic obstructive pulmonary disease | 86 (5.7%) | 55 (5.2%) | 31 (6.8%) | 0.210 |
| Peptic ulcer | 43 (2.8%) | 31 (2.9%) | 12 (2.6%) | 0.758 |
| Pulmonary infection | 154 (10.2%) | 109 (10.3%) | 45 (9.9%) | 0.818 |
| IVUS-guided PCI | 285 (18.8%) | 203 (19.2%) | 82 (18.1%) | 0.608 |
| OCT-guided PCI | 57 (3.8%) | 31 (2.9%) | 26 (5.7%) | 0.009 |
| FFR-guided PCI | 67 (4.4%) | 47 (4.4%) | 20 (4.4%) | 0.974 |
| Transfer to CCU | 200 (13.2%) | 149 (14.1%) | 51 (11.2%) | 0.134 |
| Left main stem lesion | 309 (20.4%) | 230 (21.7%) | 79 (17.4%) | 0.055 |
| Multivessel disease | 662 (43.8%) | 468 (44.2%) | 194 (42.7%) | 0.589 |
| Femoral access | 18 (1.2%) | 11 (1.0%) | 7 (1.5%) | 0.409 |
| Intra-aortic balloon pump | 51 (3.4%) | 40 (3.8%) | 11 (2.4%) | 0.180 |
| Chronic total occlusion | 350 (23.1%) | 251 (23.7%) | 99 (21.8%) | 0.418 |
| LV | 48.9 (46.0, 54.0) | 49.0 (46.0, 54.0) | 48.0 (46.0, 54.0) | 0.102 |
| LA | 37.0 (34.4, 40.0) | 37.0 (34.0, 41.0) | 37.0 (35.0, 40.0) | 0.971 |
| RV | 22.00 (20.00, 23.00) | 22.00 (20.00, 23.00) | 22.00 (20.00, 23.00) | 0.266 |
| RA | 34 (32, 38) | 34 (32, 38) | 34 (32, 38) | 0.564 |
| IVS | 12.00 (10.00, 13.58) | 12.00 (10.00, 14.00) | 11.00 (10.00, 13.00) | 0.122 |
| LVPW | 9.00 (9.00, 10.00) | 9.28 (9.00, 10.00) | 9.00 (9.00, 10.00) | 0.292 |
| AAO | 35 (34, 39) | 36 (34, 39) | 35 (34, 39) | 0.557 |
| EF | 61 (50, 68) | 61 (50, 68) | 62 (51, 69) | 0.243 |
| Hb | 124 (109, 139) | 123 (109, 139) | 125 (110, 138) | 0.729 |
| PLT | 180 (137, 224) | 180 (137, 223) | 178 (136, 227) | 0.848 |
| WBC | 6.72 (5.37, 8.46) | 6.76 (5.44, 8.59) | 6.51 (5.22, 8.17) | 0.011 |
| Neu | 4.37 (3.36, 5.95) | 4.45 (3.41, 6.12) | 4.13 (3.18, 5.67) | 0.005 |
| RBC | 4.14 (3.66, 4.61) | 4.15 (3.66, 4.63) | 4.12 (3.67, 4.54) | 0.431 |
| HBDH | 156 (133, 207) | 156 (133, 209) | 157 (132, 206) | 0.843 |
| TG | 1.50 (1.07, 2.15) | 1.48 (1.06, 2.09) | 1.53 (1.07, 2.32) | 0.483 |
| Glu | 7.1 (5.6, 10.0) | 7.3 (5.7, 10.1) | 6.6 (5.4, 9.3) | 0.004 |
| βHB | 0.11 (0.08, 0.20) | 0.11 (0.08, 0.21) | 0.10 (0.07, 0.18) | 0.184 |
| AG | 15.3 (11.3, 17.7) | 15.2 (11.1, 17.6) | 15.7 (11.8, 18.0) | 0.062 |
| CO2 | 22.7 (20.6, 24.5) | 22.6 (20.5, 24.5) | 22.8 (20.8, 24.5) | 0.391 |
| MCV | 93.5 (90.3, 96.9) | 93.2 (90.2, 96.7) | 94.1 (90.5, 97.4) | 0.011 |
| Monocyte | 0.54 (0.42, 0.71) | 0.54 (0.43, 0.74) | 0.52 (0.40, 0.67) | 0.002 |
| Lymphocyte | 1.36 (0.99, 1.79) | 1.36 (0.98, 1.77) | 1.36 (0.99, 1.84) | 0.424 |
| CTnT | 24 (12, 305) | 24 (11, 360) | 27 (13, 219) | 0.775 |
| Myo | 41 (28, 86) | 42 (28, 88) | 40 (27, 74) | 0.086 |
| CK-MB | 2 (1, 4) | 2 (1, 4) | 2 (1, 4) | 0.385 |
| BNP | 611 (151, 2,305) | 643 (153, 2,360) | 546 (150, 2,139) | 0.455 |
| AST | 23 (18, 40) | 23 (17, 40) | 23 (18, 42) | 0.741 |
| ALT | 22 (14, 42) | 21 (14, 42) | 22 (14, 42) | 0.641 |
| LDH | 210 (174, 324) | 209 (173, 338) | 212 (176, 300) | 0.744 |
| ALB | 41 (37, 44) | 41 (36, 44) | 42 (38, 44) | 0.040 |
| LDLC | 1.76 (1.22, 2.40) | 1.70 (1.16, 2.38) | 1.85 (1.34, 2.54) | 0.002 |
| UA | 340 (265, 429) | 344 (264, 430) | 339 (270, 418) | 0.496 |
| eGFR | 65 (42, 85) | 65 (41, 85) | 65 (44, 86) | 0.292 |
| Na | 138.7 (136.3, 140.7) | 138.7 (136.1, 140.8) | 138.7 (136.6, 140.6) | 0.292 |
| K | 4.00 (3.67, 4.30) | 3.99 (3.64, 4.29) | 4.01 (3.72, 4.31) | 0.200 |
| Ca | 2.23 (2.11, 2.32) | 2.23 (2.10, 2.32) | 2.24 (2.14, 2.32) | 0.142 |
| cyc | 1.10 (0.75, 1.44) | 1.10 (0.75, 1.43) | 1.11 (0.77, 1.45) | 0.539 |
| Total protein | 67 (61, 71) | 66 (61, 70) | 67 (62, 71) | 0.076 |
| Total cholesterol | 9.5 (6.9, 12.4) | 9.7 (6.9, 12.5) | 9.2 (6.9, 11.8) | 0.091 |
| HBA1C | 6.60 (5.90, 8.40) | 6.60 (5.90, 8.40) | 6.60 (5.90, 8.30) | 0.976 |
| PCT | 0.02 (0.02, 0.03) | 0.02 (0.02, 0.03) | 0.02 (0.02, 0.03) | 0.865 |
| DDimer | 0.49 (0.27, 1.04) | 0.51 (0.27, 1.07) | 0.44 (0.26, 0.97) | 0.279 |
| INR | 1.01 (0.95, 1.08) | 1.01 (0.95, 1.09) | 1.01 (0.95, 1.07) | 0.296 |
| APTT | 28.2 (26.3, 30.3) | 28.2 (26.3, 30.2) | 28.0 (26.2, 30.5) | 0.770 |
| Fib | 2.85 (2.23, 3.63) | 2.85 (2.22, 3.63) | 2.86 (2.24, 3.63) | 0.793 |
| FDP | 2.50 (2.50, 2.51) | 2.50 (2.50, 2.60) | 2.50 (2.50, 2.50) | 0.433 |

HR, heart rate; ACS, acute coronary syndrome; CKD, chronic kidney disease; COPD, chronic obstructive pulmonary disease; IVUS-guided PCI, intravascular ultrasound-guided percutaneous coronary intervention; OCT-guided PCI, optical coherence tomography-guided percutaneous coronary intervention; FFR-guided PCI, fractional flow reserve-guided percutaneous coronary intervention; CCU, coronary care unit; IABP, intra-aortic balloon pump; CTO, chronic total occlusion; LV, left ventricle; LA, left atrium; RV, right ventricle; RA, right atrium; IVS, interventricular septum; LVPW, left ventricular posterior wall; AAO, ascending aorta; EF, ejection fraction; Hb, hemoglobin; PLT, platelet; WBC, white blood cell; Neu, neutrophil; RBC, red blood cell; HBDH, hydroxybutyrate dehydrogenase; TG, triglycerides; Glu, glucose; βHB, beta-hydroxybutyrate; AG, anion gap; CO2, carbon dioxide; MCV, mean corpuscular volume; CTnT, cardiac troponin T; Myo, myoglobin; CK-MB, creatine kinase-MB; BNP, B-type natriuretic peptide; AST, aspartate aminotransferase; ALT, alanine aminotransferase; LDH, lactate dehydrogenase; ALB, albumin; LDLC, low-density lipoprotein cholesterol; UA, uric acid; eGFR, estimated glomerular filtration rate; Na, sodium; K, potassium; Ca, calcium; HbA1c, hemoglobin A1C; PCT, procalcitonin; INR, international normalized ratio; APTT, activated partial thromboplastin time; Fib, fibrinogen; and FDP, fibrin degradation products.
